# Effects of *Andrographis paniculata* and *Orthosiphon stamineus* Extracts on the Glucuronidation of 4-Methylumbelliferone in Human UGT Isoforms

**DOI:** 10.3390/molecules15053578

**Published:** 2010-05-14

**Authors:** Sabariah Ismail, Nur Aziah Hanapi, Mohd Rohaimi Ab Halim, Verawan Uchaipichat, Peter I. Mackenzie

**Affiliations:** 1 Centre for Drug Research, Universiti Sains Malaysia, 11800, Penang, Malaysia; 2 Department of Clinical Pharmacology, Flinders University, Adelaide, Australia; E-Mails: verawan.uchaipichat@flinders.edu.au (V.U.); peter.mackenzie@flinders.edu.au (P.I.M.)

**Keywords:** UGT, inhibition, herbal extracts, *Orthosiphon stamineus*, *Andrographis paniculata*

## Abstract

The effects of *Andrographis paniculata* and *Orthosiphon stamineus *extracts on the *in vitro* glucuronidation of 4-methylumbelliferone (4MU) by recombinant human UGTs, UGT1A1, UGT1A3, UGT1A6, UGT1A7, UGT1A8, UGT1A10, UGT2B7 and UGT2B15 were determined. The potential inhibitory effects of both of the extracts on the activity of each of the UGT isoforms were investigated using 4MU as the substrate. Incubations contained UDP-glucuronic acid (UDPGA) as the cofactor, MgCl_2_, cell lysate of respective isoform, and 4MU at the approximate apparent K_m_ or S_50_ value of each isoform. Final concentrations of *Andrographis paniculata* and *Orthosiphon stamineus* extracts used were 0.025, 0.25, 2.5, 25 and 50 μg/mL and 0.01, 0.10, 1.0, 10 and 50 μg/mL respectively. Both extracts variably inhibited the activity of most of the isoforms in a concentration dependent manner. *Andrographis paniculata* extract was the better inhibitor of all the isoforms studied (IC_50_ 1.70 μg/mL for UGT1A3, 2.57 μg/mL for UGT1A8, 2.82 μg/mL for UGT2B7, 5.00 μg/mL for UGT1A1, 5.66 μg/mL for UGT1A6, 9.88 μg/mL for UGT1A7 and 15.66 μg/mL for UGT1A10). Both extracts showed less than 70% inhibition of UGT2B15, so the IC_50_ values were >50μg/mL. The inhibition of human UGTs by *Andrographis paniculata* and *Orthosiphon stamineus *extracts *in vitro* suggests a potential for drug-herbal extract interactions in the therapeutic setting.

## 1. Introduction

Currently it is becoming more common to consume drugs with medicinal herbs, and studies that evaluate the potential or possible interactions of therapeutic drugs and herbal medicines are therefore much needed. In Malaysia, two of the most commonly used medicinal herbs are *Andrographis Paniculata* and *Orthosiphon Stamineus*.

*Andrographis paniculata* (Burm. F.) Nees from the Acanthaceae family, indigenous to China, India and South East Asia has been traditionally used in Asia for gastric disorders, colds, influenza and other infectious diseases [1]. For the last two decades, a standardized extract of *Andrographis paniculata* has become popular in Scandinavia in treating the common cold [2]. There are also reports on *Andrographis paniculata*’s effectiveness as an immunostimulant [3] and as an anti-HIV drug [4]. 

*Andrographis paniculata* extract has been reported to inhibit rat hepatic phase I drug metabolizing enzymes such as aniline hydroxylase, *N*-demethylase and *O*-demethylase under *in vitro* and short term *in vivo* conditions [5]. An 80% hydroalcoholic extract of *Andrographis paniculata* has also been shown to increase the activity of a phase II drug metabolizing enzyme, glutathione-*S*-transferase (GST) in mice livers after treatment of *Andrographis paniculata* extract at 50 and 100 mg/kg body wt per day for 14 days [6]. A study noted that both the aqueous and the alcoholic extracts of *Andrographis paniculata* significantly increased ethoxyresorufin *O*-dealkylase and pentoxyresorufin *O*-dealkylase activities while those of methoxyresorufin *O*-dealkylase activities remain unaltered [7]. More recently, an *in vitro* study with rat and human liver microsomal hepatic cytochrome P450s showed that *Andrographis paniculata *inhibited the catalytic activities of both rat and human liver microsomal CYP1A2, CYP2C and of human liver microsomal CYP3A4 [8].

*Orthosiphon stamineus* Benth. (Lamiaceac) derives its common name ‘Misai kucing’ or cat’s whiskers from the wispy stamens of its flower, shaped like cat’s whiskers. This herbaceous shrub native to South East Asia has been used to treat urinary lithiasis, edema, fever, influenza, rheumatism, hepatitis and jaundice [9]. The methanolic extract of *Orthosiphon stamineus* had been found to exhibit cytotoxic activity against a highly liver-metastatic colon 26-L5 carcinoma cells [10]. The principal active components of *Orthosiphon stamineus* are flavonoids such as sinensetin, eupatorin, tetramethoxyflavonone and a caffeic acid derivative, rosmarinic acid [11]. There are no published reports on the effect of *Orthosiphon stamineus* on the activities of phase I and/or phase II drug metabolism.

Drug metabolizing enzymes are divided into phase I enzymes (functionalization reactions) and phase II (conjugation reactions). The phase I system is mainly comprised of the cytochrome P450 family of enzymes, which are regarded as the first defense of the body against xenobiotics. The metabolites from phase I metabolism which become more water soluble enter phase II conjugation reactions to increase water solubility of the metabolites to be easily excreted into urine or bile.

Glucuronidation is the major phase II biotransformation reaction. It involves the transfer of glucuronic acid from uridine 5’-phosphoglucuronic acid (UDPGA) to countless structurally unrelated endobiotics and xenobiotics substances possessing hydroxyl, carboxyl, amino or sulfhydryl groups, converting them to water-soluble glucuronides. A family of enzymes known as UDP-glucuronyltransferases (UGT) is responsible for this reaction. UGT isoforms have been classified to two main families UGT1 and UGT2, depending on gene structure and amino acids sequence similarities. The UGT1A and UGT2B metabolize different compounds. The UGT1A family mainly metabolizes phenolic compounds such as estrone, 2-hydroxyestrone, 4-nitrophenol, 1-naphthol, *etc.* with the involvement of bilirubin. The UGT2B family metabolizes steroid compounds such as androsterone, linoleic acid, *etc.* with the involvement of bile acids. [12]

Although glucuronidation, is increasingly recognized as a major phase II detoxification pathways in humans [13] there is less awareness of the potential interactions of herbal preparations on human UGT isoforms. The aim of this study was to establish the potential for *Andrographis paniculata *and *Orthosiphon stamineus* extracts to affect the *in vitro* glucuronidation of a marker substrate 4-methylumbelliferone (4MU), by cDNA-expressed human UGT isoforms (a panel of human recombinant UGT isoforms 1A1, 1A3,1A6, 1A7, 1A8, 1A10, 2B7 and 2B15.). 4MU is known to be metabolized by multiple human UGT isoforms, and therefore is a convenient substrate for investigating the inhibitory effects of the herbal extracts on UGT isoforms [14].

## 2. Results and Discussion

For standardization purposes, the content of the markers for both herbal extracts, polymethoxylated flavones in *Orthosiphon stamineus* and lactones such as andrographolide and neoandrographolide in *Andrographis paniculata* were determined. 

*Andrographis paniculata* extract contained as its primary constituents lactones such as andrographolide, neoandrographolide and deoxyandrographolide, along with flavonoids. Using HPLC, the percentages of andrographolide and neoandrographolide in the extract used in this study were determined to be 15.6% and 4.9%, respectively.

The methanolic extract of *Orthosiphon stamineus* is known to contain volatile oil, caffeic acid derivatives, diterpene esters, triterpin saponins, antioxidants and flavonoids [15]. In this study, the amount of three methoxylated flavones: sinensitin, eupatorin and 3’-hydroxy-5,6,7,4’-tetramethoxy-flavone and rosmarinic acid, a caffeic acid derivative in the *Orthosiphon stamineus* extract were determined. The amount of rosmarinic acid was 6.15%, 3’-hydroxy-5,6,7,4’-tetramethoxyflavone was 0.12%, sinensitin was 0.14% and eupatorin 0.31%. The contents of the marker’s (in percentage) are in agreement with a previous study [11].

Not much is known about the potential interactions of herbal extracts with UGT isoforms. Several studies had shown that medicinal herbs inhibit UGT-mediated metabolism [16,17]. Recently, a study by Katoh *et al*. [18] had shown that medicinal herbs such as Daio (*Rhei Rhizoma*), Kanzo (*Glycyrrhizae Radix*), Keihi (*Cinnamomi Cortex*) and Ogon (*Scutellariae Radix*) exhibited potent inhibition on beta-estradiol 3-glucuronidation *in vitro*.

Therefore, a series of *in vitro* experiments to study the effects of two herbal extracts commonly used in Malaysia, *Andrographis paniculata* and *Orthosiphon stamineus* on the activity of cDNA-expressed UGT isoforms were conducted. A panel of recombinant human UGT isoforms was incubated with increasing concentrations of the extracts with 4MU concentrations at the approximate apparent Km or S_50_ values for the individual isoforms (the values were determined previously by Uchaipichat *et al*. [14]). Table 1 lists the different 4MU concentrations used (corresponding to the apparent Km or S_50_ values obtained) that were used for each isoform, it also lists the different amounts of protein and incubation times employed for each isoform. These optimized conditions, vary between the isoforms but were necessary so that the rate of product formation is linear with respect to protein concentration and incubation time. 

**Figure 1:**
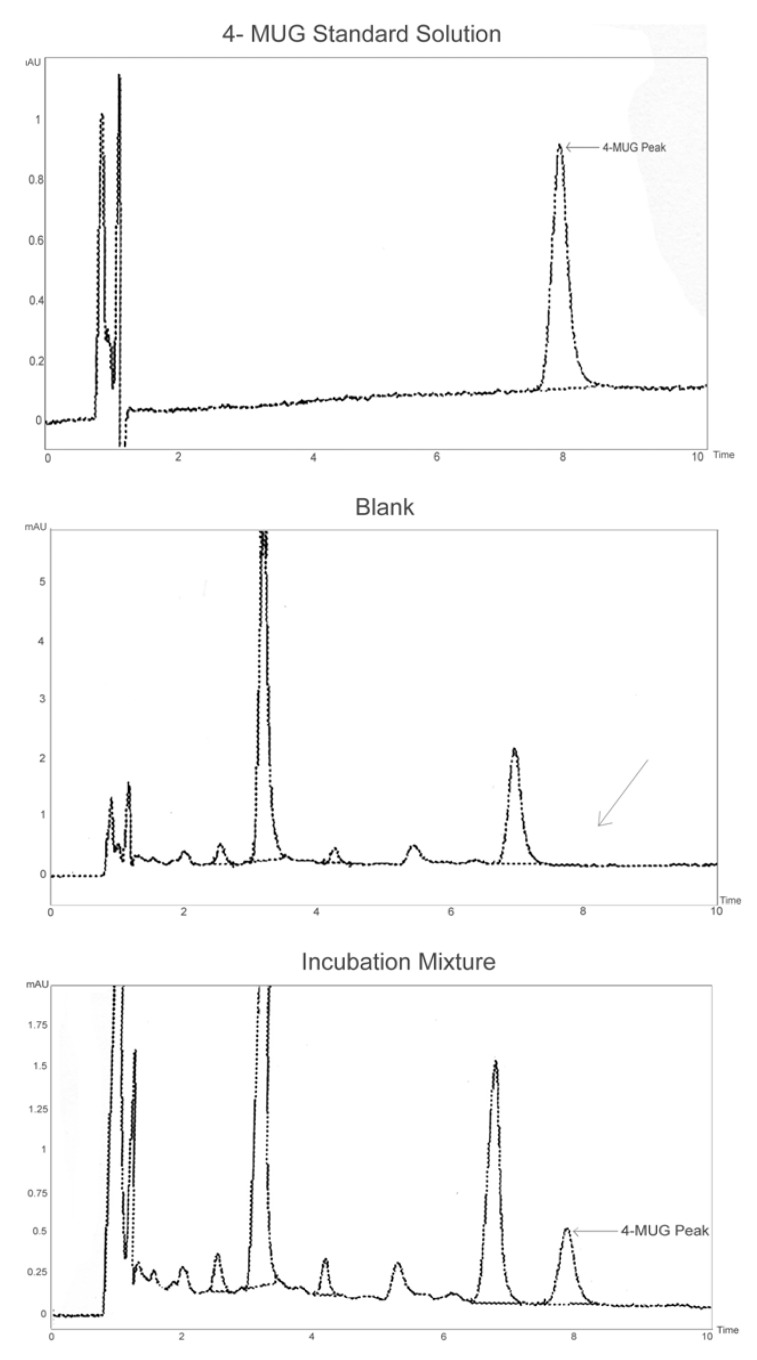
HPLC separation of 4-MU glucuronide (4-MUG) in a standard solution or in the cell lysate. In the blank (with *Orthosiphon stamineus* extract and UDPGA added but without the substrate 4MU in a standard incubation mixture)chromatogram, the arrow shows no interference where 4-MUG elutes whereas in the incubation mixture chromatogram, 4-MUG is formed by the isoform in a standard incubation mixture of 4MU, UDPGA, MgCl_2_ and the *Orthosiphon stamineus* extract in a phosphate buffer (0.1 M, pH 7.4).

HPLC chromatograms that show separation of 4MUG in a standard solution or in the cell lysate incubation mixture are presented in Figure 1. Retention times of 4-methylumbelliferone glucuronide (4MUG) and 4MU were 8.0 and 11.5 min, respectively. Concentrations of 4MUG in incubation samples were determined by comparison of peak areas to those of 4MUG standard curve with concentrations in the range 0.5–10 µM. Standard curves of 4MUG were linear in the range 0.5–10 µM, and the coefficient of variation for the slopes of 25 standard curves was 5.0%.

**Figure 2:**
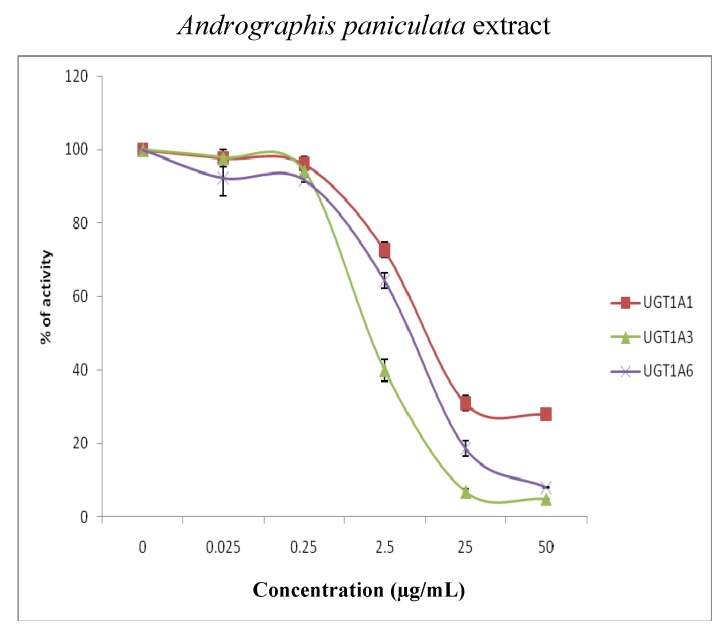

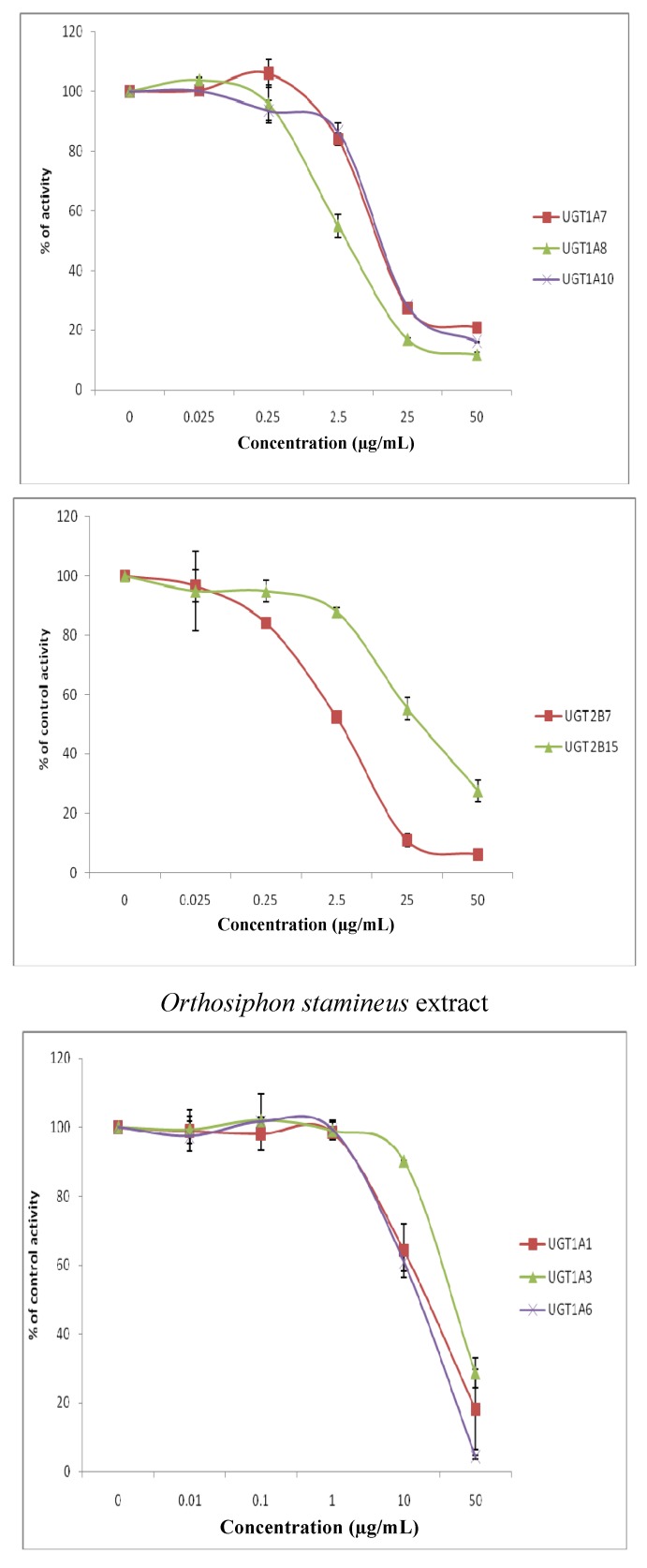

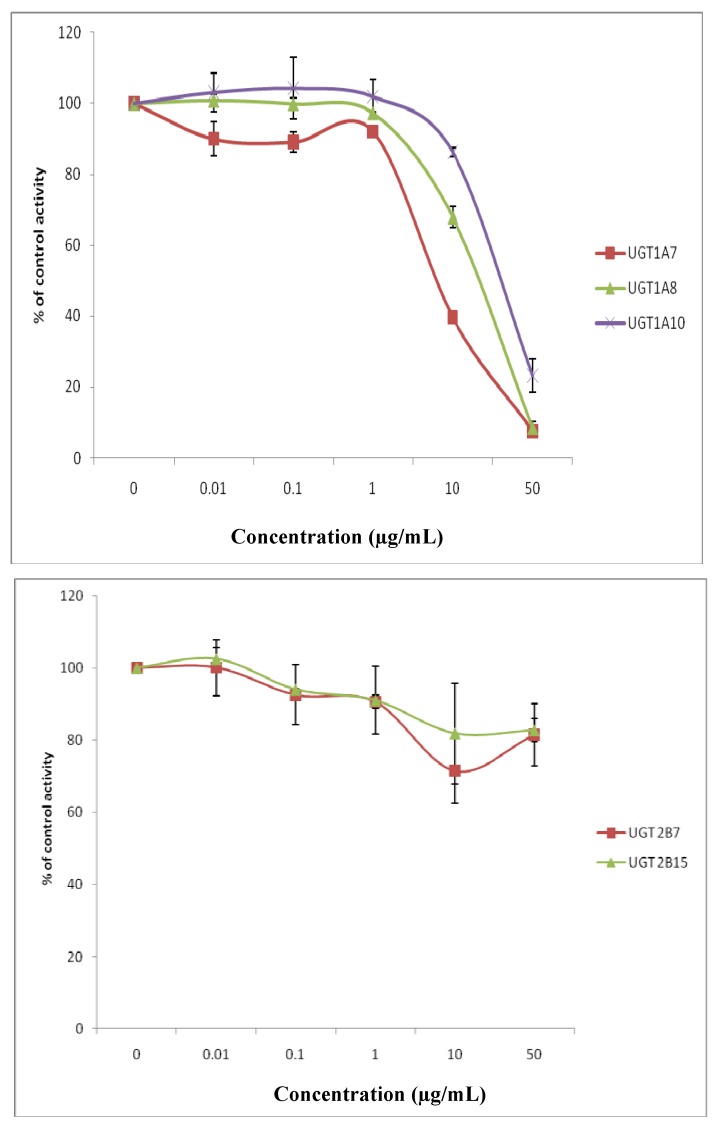
Effects of *Andrographis paniculata* extract (0.025–50 µg/mL) and *Orthosiphon stamineus* extract (0.01–50 µg/mL) on the glucuronidation of 4-methylumbelliferone in recombinant human isoforms (UGT1A1, UGT1A3, UGT1A6, UGT1A7, UGT1A8, UGT1A10, UGT2B7 and UGT2B15) presented as percentage of control. Data are expressed as means and range of two replicates.

The effects of *Andrographis paniculata* (0.025 to 50 μg/mL) and *Orthosiphon stamineus* (0.01 to 50 μg/mL) extracts on the activities of UGT 1A1, 1A3, 1A6, 1A7, 1A8, 1A10, 2B7 and 2B15 were determined at the 4MU concentration corresponding to the respective K_m_ or S_50_ values of these isoforms. Both extracts variably inhibited the activity of most of the isoforms in a concentration dependent manner (Figure 2). The data in Figure 1 were used to determine the IC_50_ values (GraphPad Prism 5) for 4MU in the presence of *Andrographis paniculata* and *Orthosiphon stamineus* extracts and the results are presented in Table 2.

**Table 2:** IC_50_ of UGT Isoforms.

Isoforms	Inhibitors (µg/mL)	
	*Andrographis paniculata *	*Orthosiphon stamineus*
UGT1A1	5.00	24.65
UGT1A3	1.70	>50
UGT1A6	5.66	30.02
UGT1A7	9.88	10.83
UGT1A8	2.57	43.39
UGT1A10	15.66	>50
UGT2B7	2.82	>50
UGT2B15	>50	>50

All UGT isoforms studied were sensitive to the inhibitory effect of *Andrographis paniculata* ethanolic extract. The inhibitory potential was in the order: UGT1A3 > UGT1A8 >UGT2B7 > UGT1A1 > UGT1A6 > UGT1A7 > UGT1A10. However, the IC_50_ was more than 50 µg/mL for UGT2B15. UGT1A3 was most sensitive to the extract with an IC_50_ of 1.70 µg/mL. UGT1A3 is human hepatic glucuronosyltransferase which glucuronidates chemicals such as tertiary amines, flavonoids and phenolic compounds. An example of a drug that is primarily cleared by UGT1A3 is propofol [19]. 

There were also considerable inhibitory effects of *Andrographis paniculata* extract on UGT1A8, UGT2B7 and UGT1A1. UGT1A8 is expressed in the intestine, but not in the liver and it is highly active against a variety of planar and bulky phenols, coumarins, flavonoids, anthaquinones, primary aromatic amines and drugs like furosemide [20]. UGT2B7 is human hepatic glucuronosyltransferase which glucuronidates endogenous compounds such as bile acid and retinoids and several xenobiotics including valproic acid, morphine, and zidovudine. It is also the most commonly listed UGT for biotransformation of the top 200 prescribed drugs in the United States of America. [21]. UGT1A1 on the other hand, is primarily responsible for the glucuronidation of bilirubin [22] and drugs such as irinotecan (a chemotherapeutic agent for the treatment of colorectal, lung, and other cancers), buprenorphine and naltrexone [23].

For *Orthosiphon stamineus* extract, the inhibitory potential was in the order: UGT1A7 > UGT1A1 > UGT1A6 > UGT1A8. The IC_50_ values for UGT1A3, UGT1A10, UGT2B7 and UGT2B15 were however higher than 50 µg/mL.

The isoform most sensitive to the inhibitory effect of *Orthosiphon stamineus* extract was UGT1A7. *Orthosiphon stamineus* at 10 µg/mL reduced the activity of UGT1A7 by 60%. UGT1A7 is a non-hepatic glucuronosyltransferase which is responsible for the glucuronidation of mycophenolic acid, a standard immunosuppressive drug [24].

There were also considerable inhibitory effects of *Orthosiphon stamineus* extract on UGT1A6, UGT1A8 and UGT1A1. UGT1A6 is the most important enzyme for the conjugation of planar molecules; it is an enzyme that transforms small lipophilic molecules, such as steroids, bilirubin, hormones, and drugs, into water-soluble, excretable metabolites. An example of a drug glucuronidated by UGT1A6 is valproic acid, a broad-spectrum antiepileptic drug.

The isoforms which were least affected by *Orthosiphon stamineus* extract were UGT2B7 and UGT2B15. *Orthosiphon stamineus* extract at 50 μg/mL had relatively minor effects (≤20% change in control activity) on UGT2B7 and UGT2B15 activities. Their IC_50 _values were higher than 50 µg/mL.

When the IC_50_ values of the *Andrographis paniculata* and *Orthosiphon stamineus* extracts on the UGT isoforms were compared to each other, it is clear that *Andrographis paniculata* is a better inhibitor than *Orthosiphon stamineus* for nearly all of the isoforms studied; UGT1A1, UGT1A3, UGT1A6, UGT1A7, UGT1A8, UGT1A10 and UGT2B7. 

It was thus of interest for us to determine if the inhibition seen with *Andrographis paniculata* was mainly due to its major active constituent; andrographolide, which is, in addition, currently being used in clinical trials [25,26]. We studied the effects of andrographolide on all isoforms except UGT2B15 since the extract had minimal effect on UGT2B15 (Figure 2). The concentration range of andrographolide that was used to evaluate its IC_50_ for all the isoforms was from 0.025 to 2.5 µg/mL (the concentration range chosen so that the concentration of andrographolide was within the same range as found in the extract). 

**Figure 3:**
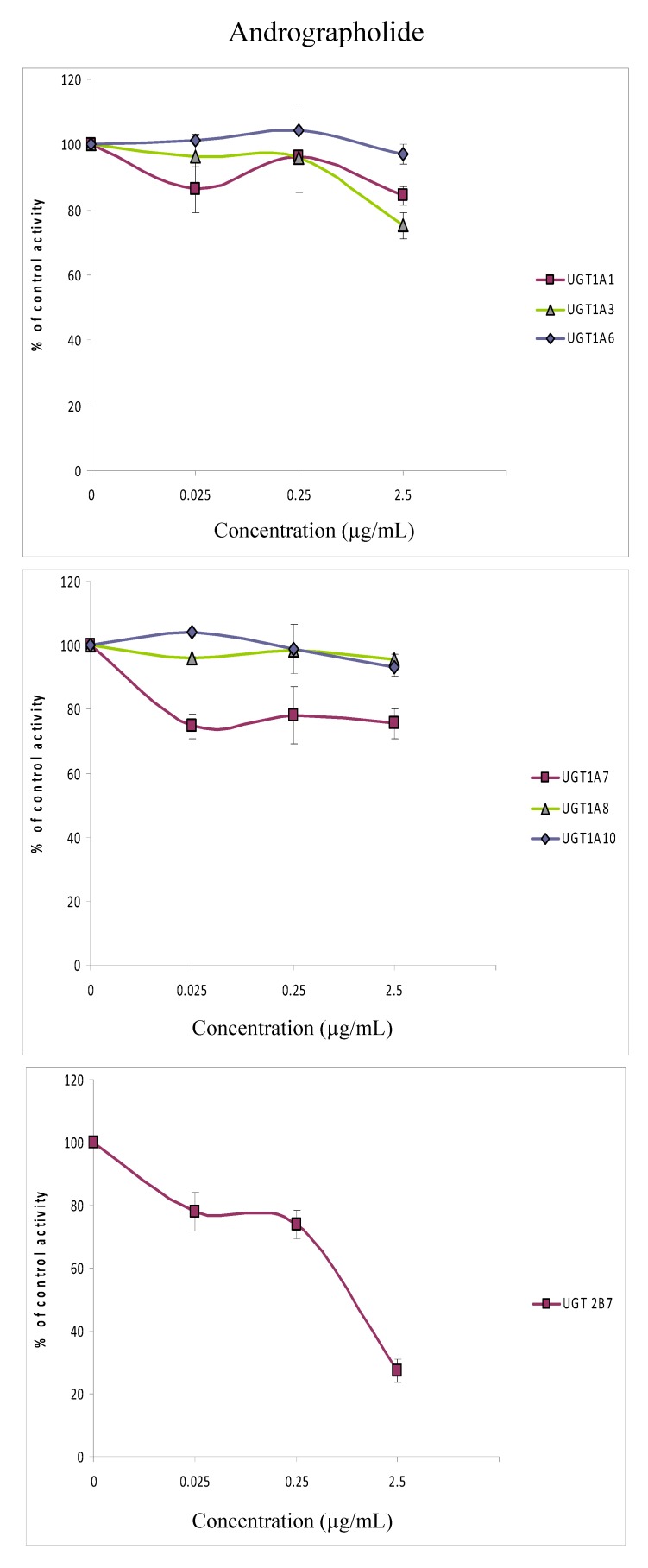
Effects of andrographolide (0.025–2.5 μg/mL) on the glucuronidation of 4-methylumbelliferone in recombinant human isoforms (UGT1A1, UGT1A3, UGT1A6, UGT1A7, UGT1A8, UGT1A10, UGT2B7) presented as percentage of control. Data are expressed as means and range of two replicates.

Using this concentration range, andrographolide did not inhibit any of the UGT1A isoforms tested. Although the activity of UGT2B7 was decreased to 27% at 2.5 µg/mL, the IC_50_ value was >50 µg/mL. The results suggest that andrographolide would not be a major contributor to the observed UGT isoforms inhibition by *Andrographis paniculata *extract. The IC_50_ values for the other isoforms; UGT1A3 and UGT1A8 could not be determined accurately as the inhibition was less than 70% at the highest concentration tested.

Since UGT2B7 is an important isoform that metabolizes many endogenous compounds and xenobiotics, and since andrographolide has been subjected to clinical trials for treatment against HIV [24] and acute upper respiratory tract infection [25] the inhibition of UGT2B7 by andrographolide in this study may be of importance clinically. 

## 3. Experimental

### 3.1. Materials

4MU, 4MU-β- D-glucuronide (4-MUG), UDP-glucuronic acid (UDPGA, trisodium salt) were purchased from Sigma-Aldrich (Sydney, New South Wales, Australia). Dried aerial parts of *Andrographis paniculata* was obtained from Landward Engineering Sdn Bhd (Melaka, Malaysia). *Orthosiphon stamineus* spray-dried extract was supplied by Chemical Engineering Pilot Plant UTM (CEPP, UTM) (Skudai, Malaysia). Standard samples of andrographolide, neoandrographolide, sinensetin (SEN), eupatorin (EUP), 3’-hydroxy-5,6,7,4’-tetramethoxyflavone (TMF) and rosmarinic acid (RA) were purchased from Indofine Chemical Co. (Hillsborough, NJ, USA). Other reagents and organic solvents were of analytical reagent grade.

### 3.2. Preparation of Andrographis paniculata extract

Dried and powdered aerial parts of *Andrographis paniculata* (1 kg) was extracted with 95% ethanol at 60 °C in a Soxhlet extractor. The ethanol extract, after concentration, (160 g of the ethanol extract are obtained from 1 kg starting material) was then analysed by HPLC. The extract was dissolved in distilled water to prepare a 5 mg/mL stock solution. Serial dilutions (0.1–1000 μg/mL) of the stock solution were then prepared.

### 3.3. HPLC analysis for the determination of andrographolide and neoandrographolide percentages in Andrographis paniculata extract

HPLC analysis was performed according to the methods of [27]. Compounds were separated using a reversed phase C18 Waters Bondapak column with the mobile phase of acetonitrile-water (70:30, v/v). Percentages of andrographolide and neoandrographolide were determined using calibration curves of the reference compounds.

### 3.4. Preparation of Orthosiphon stamineus extract

The extract used was spray-dried 50% methanolic powder of *Orthosiphon stamineus* (UTMSDE 06b). The powder form extract was dissolved in distilled water to make up a stock solution of 5 mg/mL. Serial dilutions of the stock solution from 0.01 μg/mL to 1000 μg/mL were prepared in distilled water.

### 3.5. HPLC analysis for the determination of sinensetin, eupatorin, 3’-hydroxy-5,6,7,4’-tetramethoxy-flavone and rosmarinic acid percentages in Orthosiphon stamineus extract

HPLC analysis was performed using an Agilent Technologies series 1100 system/series equipped with an automatic injector, a column oven, and a diode array UV detector according to the methods by [11]. All compounds were separated using a reversed phase C_18_, Lichrosorb column with the mobile phase of methanol-water-tetrahydrofuran (45:50:5 v/v). Quantification of the compounds in the extract was done using standard calibration curves established by plotting the areas of peaks against different concentrations of pure reference compounds. 

### 3.6. Expression of UGT proteins

Details of the UGT 1A3, 1A6, 1A8, 1A9, 1A10 and 2B7 cDNAs used here have been reported previously [28,29,30,31,32]. cDNAs encoding UGT1A1, 1A7 and 2B15 were polymerase chain reaction-amplified from CaCo2 or HepG2 cells or from a human cDNA library, and the identity of the coding regions was confirmed by sequence analysis. The individual UGT cDNAs were stably expressed in a human embryonic kidney cell line (HEK293) as described previously by [33] and [34]. Transfection of the cells with individual UGT cDNAs and the subsequent preparation of the cell lysates were as described previously [14].

### 3.7. 4MU glucuronidation assays

4MU glucuronidation was measured using a previously published procedure [35]. The amount of protein, the incubation time and the concentration of 4MU used in the measurement of 4MU glucuronidation were as described in [34] Briefly, incubations (total volume 200 µL) contained UDPGA (5 mM), MgCl2 (5 mM), cell lysate (protein concentrations given in Table 1), 4MU (concentrations also given in Table 1) and phosphate buffer (0.1 M, pH 7.4). After a 5-min preincubation at 37 ºC in a shaking water bath, reactions were initiated by the addition of UDPGA. Incubation times for each isoform activity are shown in Table 1. Reactions were terminated by the addition of 24% HClO_4_ (10 μL), samples were centrifuged (5,000*g* for 10 min) and a 20 µL of the supernatant fraction was injected into the HPLC column. 

HPLC analysis was performed using an Agilent 1100 series instrument fitted with a Security Guard C_18_ cartridge (4 × 3 mm, Phenomenex, Sydney, Australia) and a NovaPak C_18_ column (3.9 × 150 mm; Waters Associates, Milford, MA, USA). Analytes were separated using a linear gradient with a flow rate of 1 mL/min. Initial conditions were 96% 10 mM triethylamine/perchloric acid, pH 2.5 + 5% (mobile phase A) and 4% acetonitrile (mobile phase B). The proportion of mobile phase B was increased to 40% over 10 min. Column eluant was monitored by UV absorbance at 316 nm. 

### 3.8. Inhibition of 4MU glucuronidation by Andrographis paniculata and Orthosiphon stamineus extracts

*Andrographis paniculata* and *Orthosiphon stamineus* extracts were screened as inhibitors of UGT isoform activities using 4MU as the substrate. Incubations were performed as described above with 4MU concentrations at the approximate apparent K_m_ or S_50_ value of each isoform. Final concentrations of *Andrographis paniculata* and *Orthosiphon stamineus* extracts used in screening experiments were 0, 0.025, 0.25, 2.5, 25 and 50 μg/mL and 0, 0.01, 0.10, 1.0, 10 and 50 μg/mL respectively. All incubations were performed in duplicate; data points represent the mean (<10% variance) of the duplicate measurements. IC_50_ analysis was done using GraphPad Prism 5 (Version 5.01, GraphPad Software, Inc., USA).

### 3.9. Inhibition of 4MU glucuronidation by andrographolide

To determine if the major constituent of *Andrographis paniculata, *andrographolide is responsible for the inhibition of the UGT isoforms, andrographolide was screened as an inhibitor of UGT isoform activities using 4MU as the substrate. All isoforms except UGT2B15 was screened for inhibition. Incubations were performed as described in section 3.8 with final concentrations of andrographolide used in screening experiments ranged from 0, 0.025, 0.25, and 2.5 μg/mL.

**Table 1:** Cell lysate protein amount, incubation time, and substrate concentration used for the inhibition of 4-methylumbelliferone (4MU) glucuronidation by *Andrographis paniculata* and *Orthosiphon stamineus* extracts.

Isoform	4MU		
	Protein amount	Incubation time	Concentration
	*µg/incubation*	*min*	*µM*
1A1	67	120	100
1A3	33	75	1000
1A6	0.5	30	100
1A7	1.67	10	15
1A8	10	30	750
1A10	10	30	30
2B7	50	120	350
2B15	167	120	300

## 4. Conclusions

The studies reported here document the potential of *Andrographis paniculata* and *Orthosiphon stamineus* extracts to inhibit human UGT enzymes *in vitro*. *Andrographis paniculata* extract inhibited both the UGT1A and UGT2B isozymes whereas *Orthosiphon stamineus* extract was more selective in inhibiting UGT1A isozymes compared to UGT2B isozymes. *Andrographis paniculata *extract is a better inhibitor than *Orthosiphon stamineus* extract for UGT1A1, UGT1A3, UGT1A6, UGT1A7, UGT1A8, UGT1A10 and UGT2B7. The major constituent of *Andrographis paniculata*, andrographolide, would not be a major contributor for the inhibition by the extract. Whether the observed potency of these herbal extracts *in vitro* can be interpreted as having potential relevance in humans via pharmacokinetic drug-drug interactions requires further investigations.
